# Portfolio management based on value distribution reinforcement learning algorithm

**DOI:** 10.3389/frai.2025.1709493

**Published:** 2026-01-30

**Authors:** Yan Yang, Tian Wang, Yiding Fu, Jingna Huang, Dong Zhou

**Affiliations:** 1 Strategic Development Department Southern Power Grid Capital Holding Co., Ltd., Guangzhou, China; 2 Southern Power Grid Financial Leasing Co., Ltd., Guangzhou, China; 3 Southern Power Grid Private Fund Management Co., Ltd., Southern Power Grid Jianxin Fund Management, Guangzhou, China

**Keywords:** portfolio optimization, reinforcement learning, value distribution risk management, quantitative finance, actor-critic algorithm

## Abstract

**Introduction:**

In the face of high uncertainty and complexity in financial markets, achieving portfolio return maximization while effectively controlling risk remains a critical challenge.

**Methods:**

We propose a novel portfolio management framework based on the value distribution maximum entropy actor-critic (VD-MEAC) reinforcement learning algorithm. We establish a framework where the agent’s actions represent portfolio weight adjustments and stock factors serve as state observations. For risk management, the critic network learns the complete distribution of future returns. For return enhancement, we incorporate entropy regularization.

**Results:**

We conduct extensive experiments using real market data from the Chinese stock market. Results demonstrate that our VD-MEAC strategy achieves an average return of 2.490 and an average Sharpe ratio of 2.978, significantly outperforming benchmark strategies.

**Discussion:**

These results validate the effectiveness of our approach in practical portfolio management scenarios.

## 1 Introduction

Portfolio management remains one of the most challenging problems in financial mathematics and quantitative investment, requiring sophisticated approaches to balance return maximization against risk minimization in highly complex and non-stationary market environments (Rezaei and Nezamabadi-Pour, 2025; Sattar et al., 2025; Xu, 2025). Traditional portfolio optimization methods, from Markowitz’s mean–variance framework to various factor models, often rely on restrictive assumptions about return distributions and market behavior that may not hold in practice (Li and Hai, 2024). With the advancement of artificial intelligence and the increasing availability of high-dimensional financial data, reinforcement learning (RL) has emerged as a promising approach to portfolio management, enabling the development of adaptive investment strategies through interaction with financial markets (Jiang et al., 2024).

In practice, prior studies have applied RL to portfolio management from different perspectives. For instance, Day et al. (2024) employed policy gradient algorithms to build trading frameworks, while Fu and Huang (2025) used Q-learning to design an intelligent portfolio management system. However, these works relied on shallow neural networks, which are insufficient to handle the increasing complexity of financial markets. With the development of reinforcement learning theory, the Actor-Critic (AC) framework, which combines the benefits of both value-based and policy-based methods, has been introduced into quantitative investment. Specifically, Kitchat et al. (2024) applied the deterministic policy gradient (DPG) method to allocate a set of cryptocurrency weights and proposed a model-free convolutional network for feature extraction. Building on DPG, Cui et al. (2024) designed a state-augmentation approach to address data heterogeneity. In addition, Cheng and Sun (2024) applied the deep deterministic policy gradient (DDPG) algorithm within the AC framework to portfolio problems, while Belyakov and Sizykh (2024) introduced a proximal optimization method under the AC framework to handle portfolio optimization with transaction costs. Furthermore, Pippas et al. (2025) integrated fuzzy representations with the AC framework and proposed an adaptive fuzzy reinforcement learning approach.

Recent applications of deep reinforcement learning in portfolio management have demonstrated promising results but continue to face critical challenges (Junfeng et al., 2024). First, conventional RL algorithms typically optimize for expected returns using point estimates, which fail to capture the full uncertainty inherent in financial returns and can lead to risk-seeking behavior unsuitable for investment applications (Betancourt and Chen, 2021; Wu et al., 2021; Jang and Seong, 2023). Second, most existing approaches suffer from overestimation bias in value functions, potentially resulting in overly aggressive investment strategies and substantial drawdowns during market downturns (Aminifar et al., 2022; Koratamaddi et al., 2021). Third, the exploration-exploitation tradeoff in financial markets presents a unique challenge, as insufficient exploration may lead to strategies that perform well historically but fail to adapt to changing market conditions (Teoh et al., 2021; Pallathadka et al., 2023).

To address these limitations, we propose a Value Distribution Maximum Entropy Actor-Critic (VD-MEAC) framework that fundamentally reimagines the application of reinforcement learning to portfolio management. Our framework makes three key innovations: (1) Instead of modeling expected returns, our Critic network learns the entire distribution of future returns, providing a more comprehensive risk assessment; (2) We implement a novel mechanism to filter out overconfident decision information in the value distribution, explicitly reducing overestimation risk; and (3) We incorporate maximum entropy reinforcement learning principles to encourage strategy diversification and robust exploration of the investment action space.

The remainder of this paper is organized as follows: Section II formulates the portfolio management problem within a reinforcement learning framework. Section III introduces our VD-MEAC algorithm, detailing its theoretical foundations and implementation. Section IV presents experimental results on real market data. Section V discusses the implications of our findings and concludes the paper.

## 2 Reinforcement learning framework for portfolio optimization

### 2.1 Portfolio problem description

Portfolio optimization involves the adjustment of asset weights by investors seeking to maximize utility at the end of an investment period (Du and Ghavidel, 2022). This problem can be expressed in the following optimization form:


$$\max_{x}[u(W(x,p(\varepsilon )))]\;$$ (1)

where x represents the investor’s trading strategy (i.e., the vector of asset weights), u(·) is the utility function, W(·) denotes the terminal wealth value, *ε* represents random factors, and p(ε) denotes asset prices.

To rigorously describe the problem, we make the following assumptions about investors and the financial environment (Jin et al., 2024; de López Prado et al., 2025):

- Investors are risk-neutral, meaning the utility function is linear.
- The asset pool consists of a fixed set of *N* risky assets and one risk-free asset, with no addition of new risky assets during the investment period.
- No minimum trading unit exists, meaning assets can be infinitely divisible.
- Trading prices are closing prices for each period, without consideration of bid-ask spreads.
- Trading costs can be represented as proportional costs.

For the optimal decision problem in Equation 1, we can employ a reinforcement learning framework for the solution. Reinforcement learning is built upon a Markov Decision Process (*S*, *A*, *R*, *ρ*, *γ*), where S represents the state space, A is the action space, R denotes the reward function, ρ is the state transition matrix (dependent on the specific policy π and the environment), and γ is the reward discount factor.

**Figure 1:**
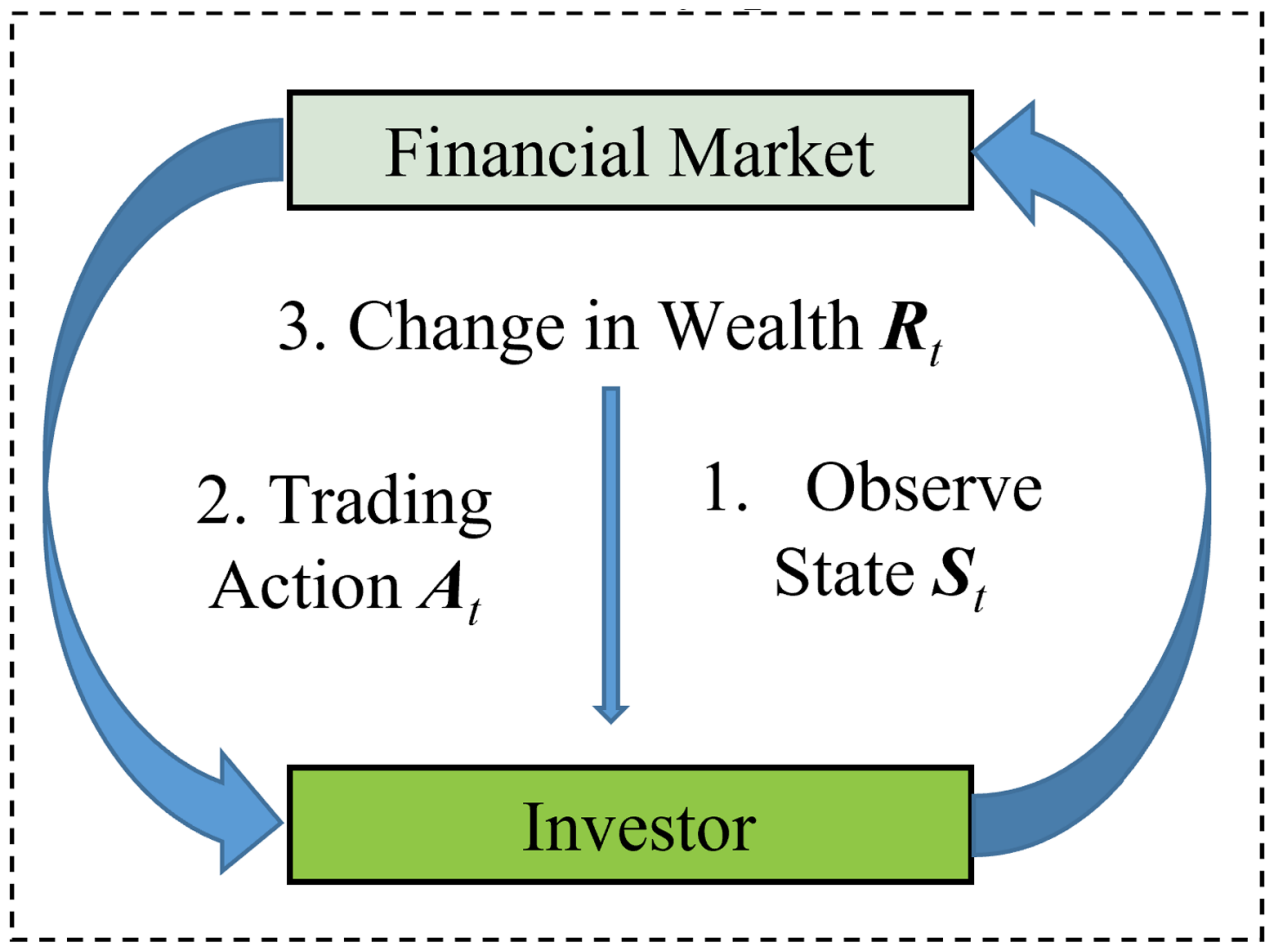
Reinforcement learning portfolio framework.

As shown in Figure 1, investors observe the state information from the financial market, take actions to adjust weights, and the financial environment provides rewards in the form of portfolio gains or losses. The ultimate goal of reinforcement learning is to train an investor that acts based on long-term benefits rather than myopic behavior.

### 2.2 Reinforcement learning framework design

State space design. Investors need to observe the state information of the financial market to make trading decisions. In this study, we choose to describe the financial market using factor information (Dong et al., 2024). The state $S_{t}$ can be represented as:


$$S_{t}=[\begin{array}{cccc} s_{01}^{t} & s_{02}^{t} & \ldots & s_{0n}^{t}\\ s_{11}^{t} & s_{12}^{t} & \ldots & s_{1n}^{t}\\ \vdots & \ddots & \vdots & \vdots \\ s_{N1}^{t} & s_{N2}^{t} & \ldots & s_{\mathrm{Nn}}^{t} \end{array}]$$ (2)

where $S_{t}$ is a matrix composed of asset factor information, and $s_{\mathrm{ij}}^{t}$ represents the value of factor j for asset i at time t .

**Figure 2:**
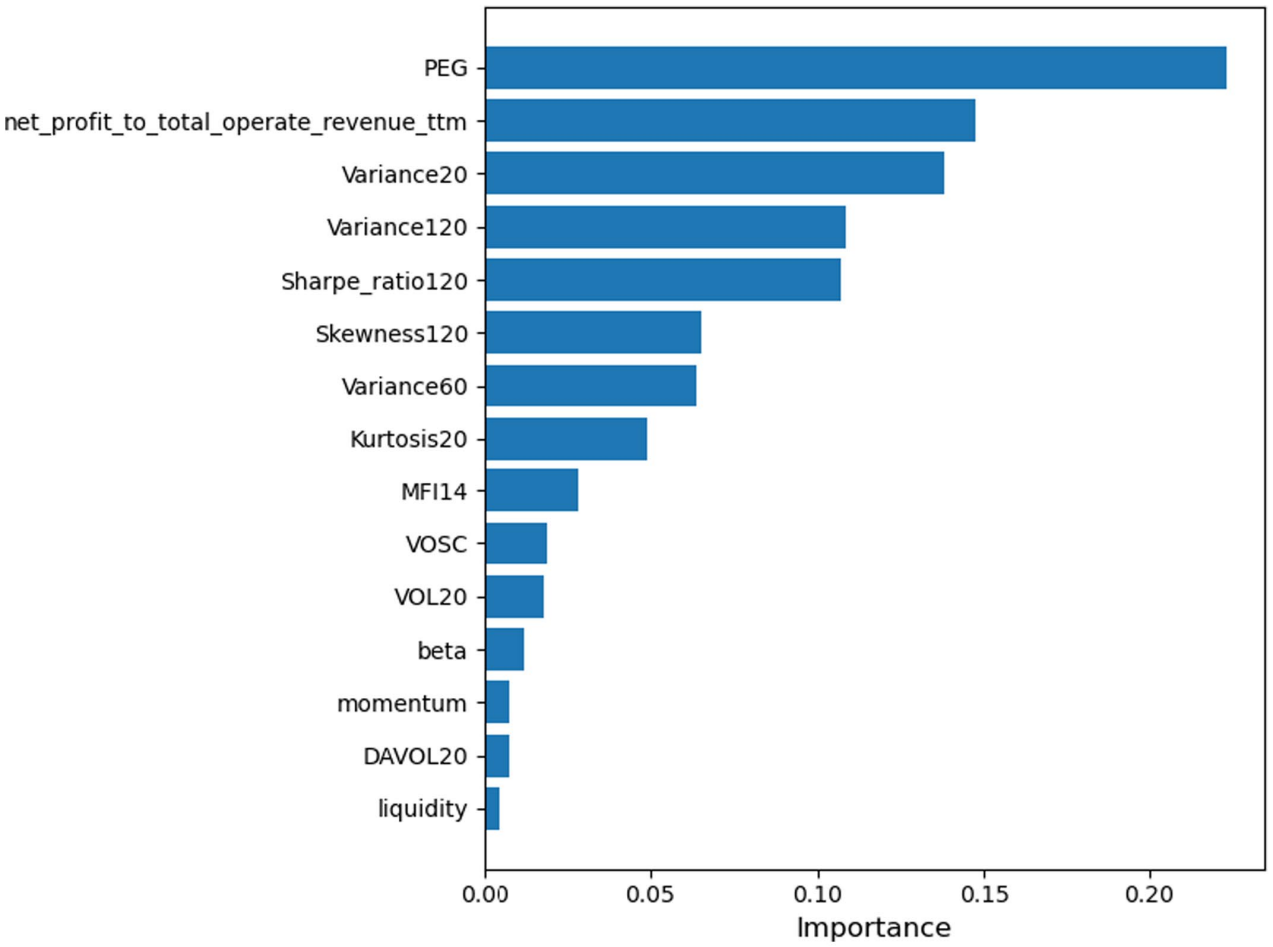
Factor importance ranking generated by LightGBM.

We use the Light Gradient Boosting Machine (LightGBM) (Gong et al., 2024) method to select important factors from the factor library. Figure 2 shows the factor importance. Investors select the top 15 important factors as observations of the financial environment before each trade, i.e., *n* = 15. The specific state information is shown in Table 1. Some factors in Table 1, such as the 20-day turnover rate, already incorporate historical information, so investors only observe the current period’s factor information rather than using a three-dimensional tensor.

**Table 1:** Specific state information.

Factor type	Factor name	Factor representation
Growth factor	Price-to-earnings growth ratio	PEG
	Net profit to total operating revenue	Net profit to total operating revenue TTM
Risk factor	120-day return variance	Variance120
	20-day return variance	Variance20
	120-day Sharpe ratio	Sharpe ratio120
	60-day return variance	Variance60
	20-day return kurtosis	Kurtosis20
	120-day return skewness	Skewness120
Technical factor	Money flow index	MFI14
Sentiment factor	20-day average turnover rate	VOL20
	Volume oscillator	VOSC
	20-day to 120-day turnover ratio	DAVOL20
Style factor	Beta, liquidity, momentum	Beta, liquidity, momentum

Action space design. Investors adjust asset weights at the beginning of each period. The action *A_t_* at time t is defined as the target portfolio weight vector *x_t_* for the end of the period, after rebalancing:


$$A_{t}=x_{t}={\left(x_{t,0},x_{t,1},\ldots ,x_{t,N}\right)}^{T}$$ (3)

where $x_{t,i}$ represents the target weight of the i-th asset (with *i* = 0 being the risk-free asset). The action space is the set of all valid weight vectors, i.e., $\sum_{i=0}^{N}x_{t,i}=1,x_{t,i}\geq 0$ (for a long-only portfolio). This constraint explicitly forbids the use of leverage or short-selling, ensuring that all performance gains are derived solely from the agent’s methodological advantages. The policy network outputs the parameters for this continuous action vector.

Reward function design. The design of the reward function is crucial. We follow the framework presented in Jiang et al. (2017) to define the reward as the one-period portfolio log return, net of transaction costs, which directly relates to maximizing terminal wealth.

Let $W_{t}$ be the portfolio value after rebalancing at time t, and $x_{t}={\left(x_{t,0},\ldots ,x_{t,N}\right)}^{T}$ be the corresponding weight vector. Let $P_{t+1}={\left(P_{t+1,0},\ldots ,P_{t+1,N}\right)}^{T}$ be the gross relative price vector from time *t* to t + 1 (i.e., ${\text{price}}_{t+1}/{\text{price}}_{t}$ ). We assume $P_{t+1,0}=1$ for the risk-free asset. The portfolio value before rebalancing at *t* + 1 is $W_{t+1}^{\prime }=W_{t}\cdot (x_{t}^{T}P_{t+1})$ .

At this point, the agent observes state $S_{t+1}$ and takes action $A_{t+1}$ to choose a new weight vector $x_{t+1}$ . Before this rebalancing, the drifted weights (due to market movement) are $x_{t}^{\prime }=(x_{t}⊙P_{t+1})/(x_{t}^{T}P_{t+1})$ , where ⊙ is element-wise multiplication.

Following Jiang et al. (2017), a proportional transaction cost *C* is incurred on the change in weights for risky assets. The portfolio value after rebalancing at *t +* 1 is: $W_{t+1}=W_{t+1}^{\prime }(1-C\cdot \sum_{i=1}^{N}\|x_{t+1,i}-x_{t,i}^{\prime }\|)$ . The one-period reward $R_{t+1}$ is then defined as the log return:


$$R_{t+1}=\ln(W_{t+1}/W_{t})=\ln((x_{t}^{T}P_{t+1})\cdot (1-C\cdot \sum_{i=1}^{N}\|x_{t+1,i}-x_{t,i}^{\prime }\|))$$ (4)

This reward function directly optimizes the cumulative log return, while correctly accounting for the friction of transaction costs as defined in Jiang et al. (2017).

## 3 Methodology

Classical reinforcement learning handles uncertainty in long-term decision processes by calculating expectations, specifically:


$$Q^{*}(s,a)=E[R(s,a)]+\gamma E[\max_{a^{\prime }}Q^{*}(s^{\prime },a^{\prime })]$$ (5)

where $Q^{*}(s,a)$ is used to evaluate the maximum expected return that the current state-action pair (s,a) can generate. In solving optimal decision problems, whether using value function-based or policy-based reinforcement learning algorithms, the accuracy of $Q^{*}(s,a)$ directly affects the algorithm’s performance. However, from the definition in Equation 5, we can see that $Q^{*}(s,a)$ only utilizes the expectation information from the distribution $\;Z^{\pi }(s,a)$ , and expectation values are easily influenced by extreme values. Furthermore, the maximization in Equation 5 and the bootstrapping TD (temporal difference) algorithm used in training inevitably produce overestimation (Zhao et al., 2024; Li et al., 2024), which in investment manifests as overconfidence, potentially leading to investment losses.

To address this, we define a stochastic policy π:S→(A) as a mapping from states to a probability distribution over actions. We then model the full distribution of the random return $Z^{\pi }(s,a)$ , which is defined as the discounted sum of future rewards: $Z^{\pi }(s,a)\cdot \sum_{k=0}^{\infty }\gamma^{k}R_{t+k+1}\;|S_{t}=s,A_{t}=a,\pi$ . The Bellman equation for this random return is:


$$Z^{\pi }(s,a)=R(s,a)+\gamma Z^{\pi }(S^{\prime },A^{\prime })$$ (6)

where $\left(S^{\prime },A^{\prime })\sim \pi (\cdot |S^{\prime }\right)$ . Our goal is to learn the distribution of $Z^{\pi }(s,a)$ , not just its expectation $Q^{\pi }(s,a)=E[Z^{\pi }(s,a)]$ .

### 3.1 Distribution function parameterization

The first issue to address is how to parameterize the distribution $Z^{\pi }(s,a)$ . We follow the Fully Parameterized Quantile Function (FQF) approach (Yang et al., 2019) to parameterize $\;Z^{\pi }(s,a)$ . According to Yang et al. (2019), any cumulative distribution function (CDF) *F_Z_* and its inverse (quantile function) $F_{Z}^{-1}$ satisfy the following relationship for the expected value $E[Z]=\int_{0}^{1}F_{Z}^{-1}\mathrm{d\omega}$ . This fundamental result allows us to represent a distribution by discretizing its quantile function. Following the FQF parameterization, we represent the return distribution as:


$$Z_{\theta ,\tau }(s,a)\cdot \sum_{i=0}^{N-1}(\tau_{i+1}-\tau_{i})\delta_{\theta_{i}(s,a)}$$ (7)

where $\delta_{\theta_{i}(s,a)}$ is the Dirac function, and τ represents the quantiles with $0=\tau_{0}<\tau_{i-1}<\tau_{i}<\tau_{N}=1$ . Our approach employs two neural networks: (1) Quantile Proposal Network *τ*: Takes state-action pair (*s*, *a*) as input and outputs adaptive quantile fractions *τ* = *τ*(*s*, *a*). (2) Quantile Value Network *θ*: Takes (*s*, *a*, *τ*) as input and outputs the quantile values *θ* = *θ*(*s*, *a*, *τ*).

For each state-action pair (s,a) , the quantile proposal network outputs quantiles τ , and the quantile value network outputs quantile values θ for each set of τ inputs.

If $F_{Z}(z)=P(Z<z)$ is the cumulative distribution function of $Z^{\pi }(s,a)$ , then its inverse function is $F_{Z}^{-1}(p)\cdot \inf\{z\in \mathbb{R}:p\leq F_{Z}(z)\}$ . According to Equation 7, we can derive the expression for the quantile values:


$$F_{Z_{\theta ,\tau }}^{-1}(\omega )=\theta_{0}+\sum_{i=0}^{N-1}(\theta_{i+1}-\theta_{i})H_{\tau_{i+1}}(\omega )$$ (8)

where $H_{\tau_{i+1}}(\omega )$ is the unit step function.

For the quantile proposal network, the closer the output quantiles τ are to the actual quantiles, the better. Thus, we define the loss function as:


$$\;W_{1}(Z,\tau )=\sum_{i=0}^{N-1}\int_{\tau_{i}}^{\tau_{i+1}}|F_{Z}^{-1}(\omega )-F_{Z}^{-1}({\overset{-}{\tau }}_{i})a|\mathrm{d\omega}$$ (9)

where ${\overset{-}{\tau }}_{i}=(\tau_{i}+\tau_{i+1})/2$ , using the Wasserstein distance.

The gradient information can be obtained by differentiating the parametric variable integral (Yang et al., 2019):


$$\frac{\partial W_{1}}{\partial \tau_{i}}=2F_{Z}^{-1}(\tau_{i})-F_{Z}^{-1}({\overset{-}{\tau }}_{i})-F_{Z}^{-1}({\overset{-}{\tau }}_{i-1})$$ (10)

Equation 10 can be simplified to avoid integral calculations, reducing the difficulty of network training. For the quantile value network, combining quantile regression with the Bellman equation, we have the TD error:


$$\delta_{\mathrm{ij}}^{t}=r_{t}+\gamma F_{Z^{\prime },w_{1}}^{-1}(\tau_{i})-F_{Z,w_{1}}^{-1}(\tau_{j})$$ (11)

where *w*_1_ denotes the target network parameters.

The loss function is chosen as the Huber quantile regression function:


$$L(s_{t},a_{t},r_{t},s_{t+1})=\sum_{i=0}^{N-1}\sum_{j=0}^{N-1}\rho_{\tau_{j}}^{\kappa }(\delta_{\mathrm{ij}}^{t})$$ (12)

where $\rho_{\tau_{j}}^{\kappa }(\delta_{\mathrm{ij}}^{t})=|\tau -I(\delta_{\mathrm{ij}}^{t}<0)|\frac{L_{\kappa }(\delta_{\mathrm{ij}}^{t})}{\kappa }$ , I(·) is the indicator function, $L_{\kappa }(\cdot )$ is the Huber loss function, and κ is the threshold value. When $|\delta_{\mathrm{ij}}|\leq \kappa$ , it is the squared error, otherwise, it is the linear error.

### 3.2 Value distribution reinforcement learning

After parameterizing the distribution $\;Z^{\pi }(s,a)$ , we need to consider how to utilize the distribution information. We adopt the Actor-Critic framework, where the distributional critic guides the actor. Our approach is based on the Soft Actor-Critic (SAC) framework (Haarnoja et al., 2018), which incorporates a maximum entropy objective to encourage exploration. Unlike SAC, our critic learns a quantile-parameterized return distribution rather than an expected *Q*-value.

High quantiles imply higher estimates of future returns for the current state-action pair (s,a) , which in finance can lead to risk due to overconfidence. Due to the overestimation problem inherent in network training, we need to discard information that might cause overestimation. We define the utilization of distribution information as:


$$Q^{\pi }(s,a)=\sum_{i=0}^{(N-1)\beta }(\tau_{i+1}-\tau_{i})F_{Z,w_{2}}^{-1}({\overset{-}{\tau }}_{i})$$ (13)

where $Q^{\pi }(s,a)$ is the guidance information from the Critic network, transmitted to the Actor network, *w_2_* denotes the quantile-value network parameters, β is the distribution information utilization coefficient with $(N-1)\beta \in {\mathbb{N}}^{+}$ . The coefficient β∈(0,1] controls the fraction of quantile information used when aggregating the learned distribution. A smaller β filters out upper-tail quantiles to mitigate overestimation. The state value function $V^{\pi }(s)$ is defined as:


$$V^{\pi }(s)=Q^{\pi }(s,a)+\mathrm{\alpha H}(\pi (\cdot |s))$$ (14)

In terms of returns, we add entropy regularization H(π(·∣s)) , using the maximum entropy principle to encourage investors to explore the action space and find more profitable trading decisions. α is the regularization coefficient; a larger α indicates stronger exploration (Zhu et al., 2024). Unlike fixed game scenarios, the financial market is a complex environment with multiple suboptimal or optimal decisions. Therefore, we prefer learning a stochastic policy to adapt to the complex financial market.

The maximum entropy objective modifies the standard reinforcement learning objective to:


$$\pi^{*}=\text{argma}x_{\pi }E_{\pi }[\sum_{t=0}^{\infty }\gamma^{t}(R(s_{t},a_{t})+\mathrm{\alpha H}(\pi (\cdot |s_{t})))]$$ (15)

This objective encourages exploration in a principled way by maximizing both the expected return and the entropy of the policy. The entropy term H(π(·∣s)) is defined as:


$$H(\pi (\cdot |s))=-E_{a\sim \pi (\cdot |s)}[\log\pi (a|s)]$$ (16)

By incorporating this entropy term, the agent is incentivized to maintain diverse action selection probabilities, preventing premature convergence to potentially suboptimal deterministic policies. This is particularly valuable in financial markets where:

- Multiple near-optimal strategies may exist.
- Market conditions change over time.
- Deterministic policies are more vulnerable to adversarial conditions.
- Exploration is necessary to discover new profitable opportunities.

### 3.3 Synergistic benefits of value distribution and maximum entropy

The true innovation of our VD-MEAC algorithm lies in the synergistic integration of value distribution learning and maximum entropy exploration. These two components complement each other in several ways:

Risk-aware exploration: The value distribution component provides rich uncertainty information that guides the entropy-based exploration toward regions with both high expected returns and manageable risk.

Robust uncertainty estimation: The maximum entropy component encourages the agent to explore diverse states, which in turn improves the quality and coverage of the learned return distributions.

Adaptive risk–return tradeoff: The combination allows for dynamic adjustment of the risk–return tradeoff based on the full distribution information rather than just point estimates.

Market regime adaptation: By maintaining policy stochasticity while capturing return distributions, the agent can quickly adapt to changing market conditions and regime shifts.

The Actor network in VD-MEAC follows a stochastic policy parameterization:


$$\pi_{\phi }(a|s)=\frac{1}{\sqrt{2\pi \sigma_{\phi }{\left(s\right)}^{2}}}\exp(-\frac{{\left(a-\mu_{\phi }(s)\right)}^{2}}{2\sigma_{\phi }{\left(s\right)}^{2}})$$ (17)

where $\mu_{\phi }(s)$ and $\sigma_{\phi }(s)$ are the mean and standard deviation of the action distribution, respectively, produced by the Actor network. This Gaussian policy allows for controlled stochasticity in portfolio weight adjustments.

### 3.4 Theoretical convergence properties

The theoretical convergence properties of VD-MEAC are founded on the established convergence guarantees of its core components. The distributional critic, based on FQF, inherits the convergence properties of distributional RL in the 1-Wasserstein metric, which is shown to be a contraction (Yang et al., 2019). The actor and its entropy-regularized objective are based on the Soft Actor-Critic framework, which provides its own policy improvement and convergence guarantees (Haarnoja et al., 2018).

**Figure 3:**
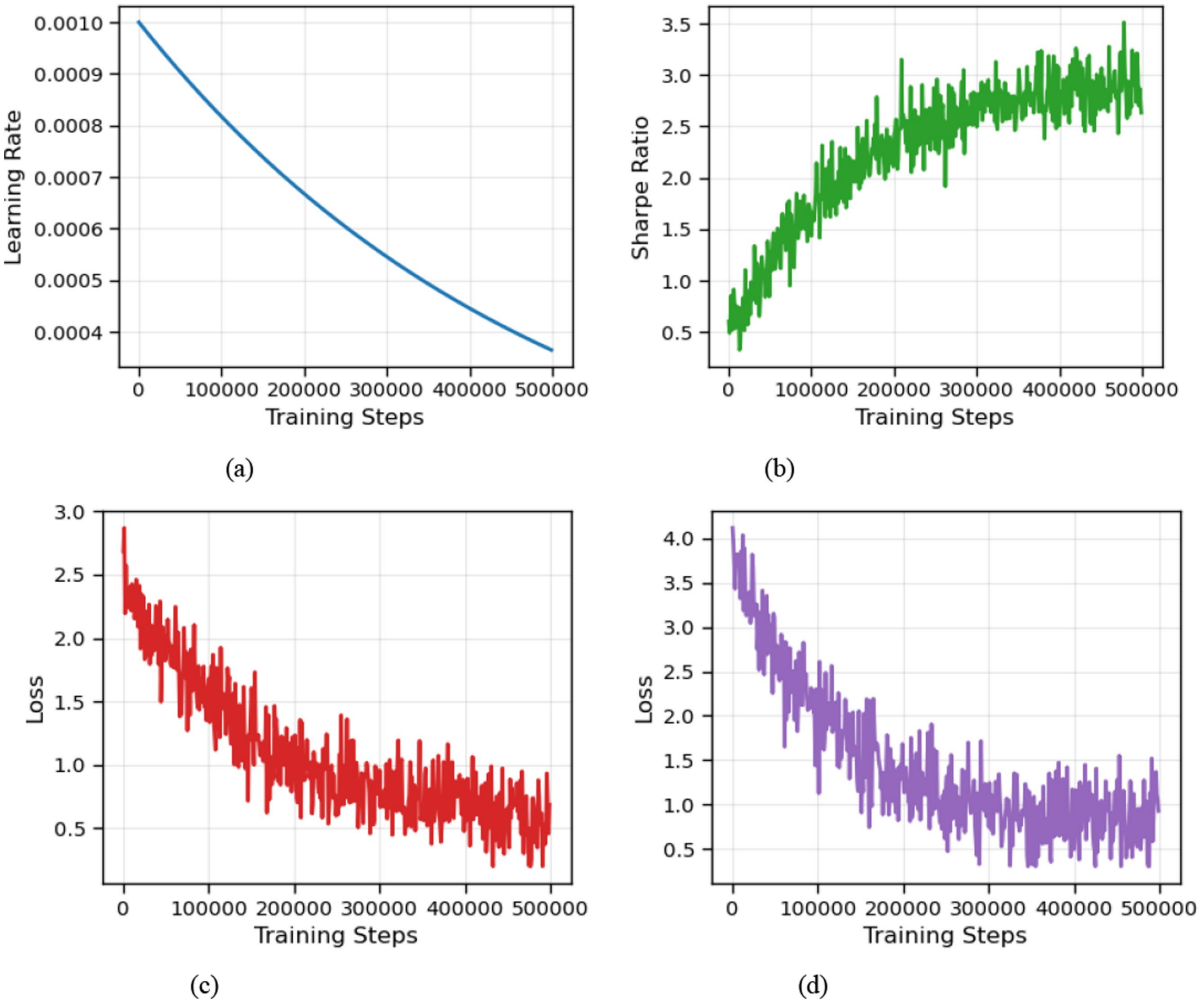
Model training results. **(a)** Learning rate, **(b)** Sharpe ratio, **(c)** Loss (Critic), and **(d)** Loss (Actor).

While a unified convergence proof for the combined VD-MEAC framework is non-trivial and left for future work, the robust empirical convergence demonstrated in our experiments (Figure 3) validates the stability and effectiveness of this synergistic approach.

**Figure 4:**
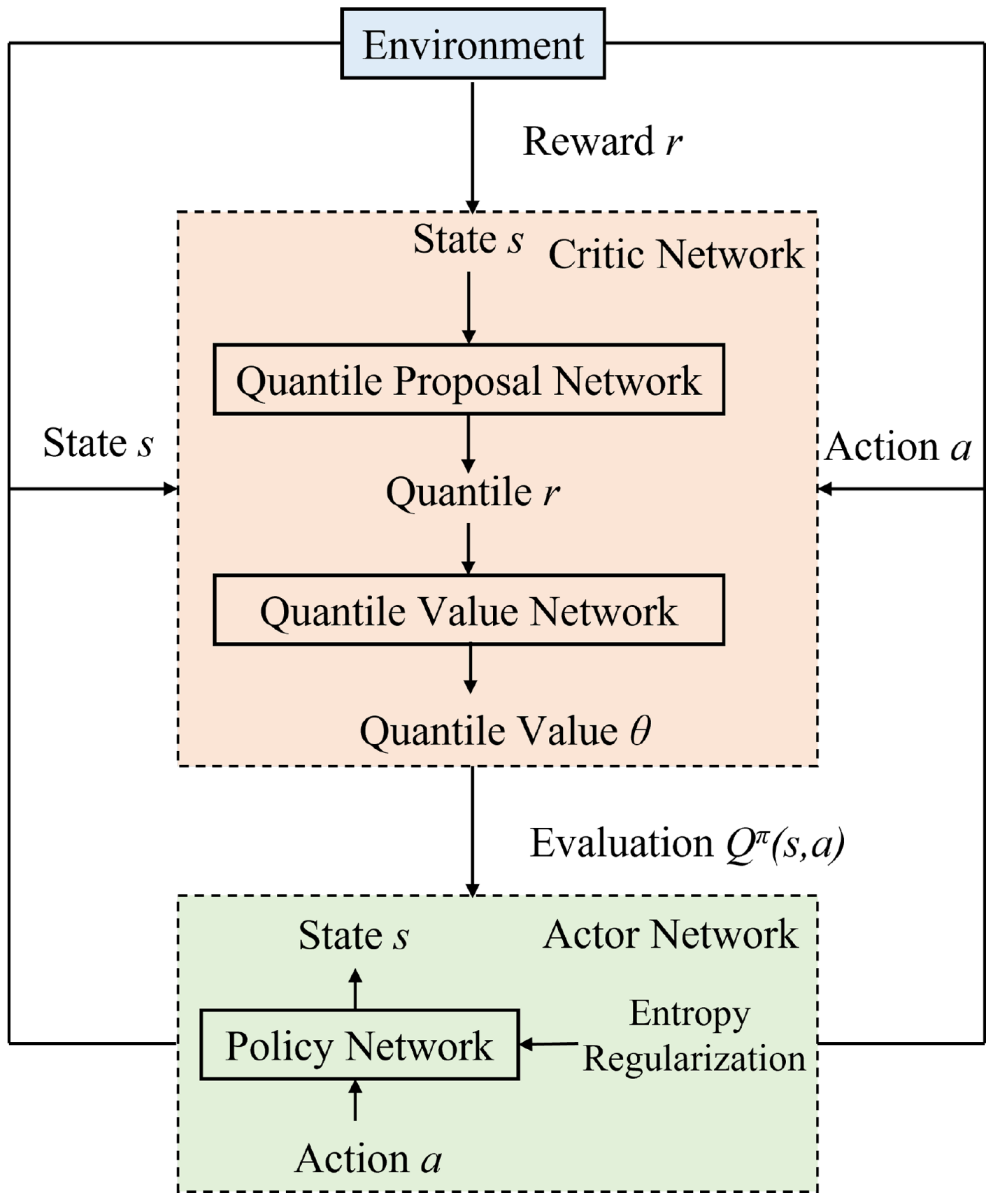
VD-MEAC algorithm flow diagram.

Algorithm 1 and Figure 4 illustrate the VD-MEAC algorithm flow. Our approach combines the strengths of distributional reinforcement learning with maximum entropy reinforcement learning to create a robust portfolio management system that effectively balances risk and return considerations.

#### ALGORITHM 1 Value distribution maximum entropy actor-critic

Initialize actor network *π**_ϕ_* with random parameters *ϕ*

Initialize quantile proposal network $\tau_{\psi }$ with random parameters ω

Initialize quantile value network $\theta_{\omega }$ with random parameters ω

Initialize target networks: ψ ‘ ← ψ , ω ‘ ←ω

Initialize replay buffer 𝓓

For each episode:

Initialize state $s_{0}$

For each time step *t*:

Observe state $s_{t}$

Sample action $a_{t}\sim \pi_{\phi }(\cdot |s_{t})$

Execute action $a_{t}$ , observe next state $s_{t+1}$ and reward $r_{t}$

Store transition $\left(s_{t},a_{t},r_{t},as_{t+1}\right)$ in 𝓓

If time to update:

1. Sample mini-batch of N transitions (s, a, r, s′) from 𝓓

2. Generate quantiles $\tau =\tau_{\psi }(s,a)$

3. Compute quantile values $\theta =\theta_{\omega }(s,a,\tau )$

4. Compute target quantiles $\tau^{\prime }=\tau_{\psi^{\prime }}(s^{\prime },a^{\prime })$ with $a^{\prime }\sim \pi_{\phi }(\cdot |s^{\prime })$

5. Compute target quantile values $\theta^{\prime }=\theta_{\omega^{\prime }}(s^{\prime },a^{\prime },\tau^{\prime })$

6. Update quantile proposal network by minimizing $W_{1}(Z,\tau )\;$ in Equation 9

7. Update quantile value network by minimizing the loss ( Equation 12 )

8. Compute filtered $Q^{\pi }(s,a)$ using Eq. 13

9. Update actor network by maximizing $Q^{\pi }(s,a)+\mathrm{\alpha H}(\pi_{\phi }(\cdot |s))$

10. Update target networks:

$\psi^{\prime }\leftarrow \tau \psi^{\prime }+(1-\tau )\psi$

$\omega^{\prime }\leftarrow \tau \omega^{\prime }+(1-\tau )\omega$

## 4 Experiments and analysis

### 4.1 Model training

To thoroughly evaluate our VD-MEAC algorithm, we designed a comprehensive experimental framework comparing against both traditional portfolio strategies and state-of-the-art reinforcement learning methods (Jeribi et al., 2024; Alzaman, 2025; Aritonang et al., 2025; Cui et al., 2025). The comparative methods are:

- Equal-weight (EW): A naive baseline that assigns equal weights to all assets, requiring no optimization but serving as a surprisingly effective benchmark in many portfolio studies.
- CSI 300 Index: A market capitalization-weighted index tracking the 300 largest stocks in China, representing the market benchmark.
- DDPG: A model-free, off-policy actor-critic algorithm using deep function approximators for continuous action spaces. DDPG combines the actor-critic approach with insights from DQN.
- TD3 (Twin Delayed DDPG): An improved version of DDPG that addresses function approximation errors by using twin critics and delayed policy updates, reducing overestimation bias.
- SAC (Soft Actor-Critic): A state-of-the-art off-policy algorithm that maximizes both expected return and entropy, encouraging exploration and robustness.

For each portfolio, we selected constituent stocks from the CSI 300 Index with minimal missing data. Any intermittent missing values (e.g., due to trading halts) within the selected stocks were filled using the forward-fill method, carrying over the last known value. This ensures continuity in price series while maintaining the most recent available information for suspended stocks. Before training, all 15 state factors were normalized using z-score normalization based on the mean and standard deviation of the training dataset (July 1, 2017–July 1, 2020). This ensures that all input features have a mean of approximately 0 and a standard deviation of 1, preventing features with larger scales from dominating the learning process. The stock list is presented in Table 2.

**Table 2:** Stock list.

Portfolio	Stock name	Stock code	Sector
A (original portfolio)	Yanzhou coal mining	600188	Energy
	YTO express	600233	Logistics
	Zhongnan construction	000961	Real estate
	China molybdenum	601958	Materials
	Shijiazhuang stone	002153	Materials
	Hundsun technologies	600446	Technology
	Tsinghua unigroup	000938	Technology
	Sinopec oilfield service	600871	Energy
	Wanhua chemical	600309	Materials
	AVIC electronics	600372	Industrials
B (financial and consumer)	China merchants bank	600036	Financials
	Ping an insurance	601318	Financials
	Industrial bank	601166	Financials
	Kweichow moutai	600519	Consumer Goods
	Yili group	600887	Consumer Goods
	Midea group	000333	Consumer Goods
	Luzhou Laojiao	000568	Consumer Goods
	China pacific insurance	601601	Financials
	CITIC securities	600,030	Financials
	China life insurance	601628	Financials
C (technology and healthcare)	Eastmoney information	300059	Technology
	Hikvision	002415	Technology
	GoerTek	002241	Technology
	iFlytek	002230	Technology
	Luxshare precision	002475	Technology
	Mindray medical	300760	Healthcare
	WuXi AppTec	603259	Healthcare
	Jiangsu Hengrui medicine	600276	Healthcare
	Tigermed consulting	300347	Healthcare
	Shenzhen Kangtai biological	300601	Healthcare

To ensure the robustness of our results and avoid selection bias, we conducted experiments on three different portfolios sampled from CSI 300 constituents:

Portfolio A (Original Portfolio): Nine stocks selected based on data completeness and sector diversity, plus one risk-free asset (government bonds).

Portfolio B (Financial & Consumer Sectors): Ten stocks from financial services and consumer goods sectors, representing defensive and stable growth characteristics.

Portfolio C (Technology & Healthcare Sectors): Ten stocks from technology and healthcare sectors, representing high-growth and innovative industries.

We implemented the VD-MEAC strategy using TensorFlow 2.4 with Python 3.8. The experiments were conducted on a high-performance computing workstation equipped with an Intel Xeon E5-2698 v4 CPU, an NVIDIA Tesla V100 GPU, and 128 GB of DDR4 RAM. The system ran on Ubuntu 20.04 LTS, ensuring a stable Linux-based environment for deep learning training. The training period spanned from July 1, 2017, to July 1, 2020, ensuring sufficient historical data to capture various market conditions. The testing period was from July 1, 2020, to September 1, 2021, encompassing both bull and bear market phases. The main parameter settings for the model are presented in Table 3, where we utilized the Adam optimizer with ReLU activation functions. The 5 × 10^5^ training steps for the VD-MEAC model took approximately 8.5 h to complete. The computational complexity of the agent at each time step is dominated by the forward passes of the actor and critic networks, which is efficient for real-time decision-making.

**Table 3:** Model main parameter settings.

Parameter name	Value
Entropy weight α	0.05
Distribution utilization β	0.75
Replay buffer capacity	1 × 10^6^
Batch size	128
Critic network architecture	[300, 200]
Actor network architecture	[64, 32]
Initial learning rate	0.001

Additionally, we used the Adam optimizer and ReLU activation functions. The model was trained for $5\times 10^{5}$ steps, with the training results shown in Figure 3. Figure 3a shows the learning rate, which incorporates decay to prevent non-convergence due to excessive learning rates. Figures 3c,d display the loss values for the Actor and Critic networks, respectively, indicating that the network training has stabilized. It’s important to note that the interpretation of loss values in reinforcement learning differs from that in deep learning; stable network training does not necessarily signify that the model has learned a profitable trading strategy. However, examining Figure 3b, we observe that the Sharpe ratio per episode increases continuously as training progresses and eventually stabilizes, suggesting model convergence.

### 4.2 Model testing

It is critical to note that all strategies compared in this section, including our own VD-MEAC, are evaluated under the strict long-only, no-leverage constraint defined in Section II-B. The superior performance of VD-MEAC is therefore derived entirely from its methodological advantages in risk modeling and exploration, not from financial engineering or hidden leverage.

**Figure 5:**
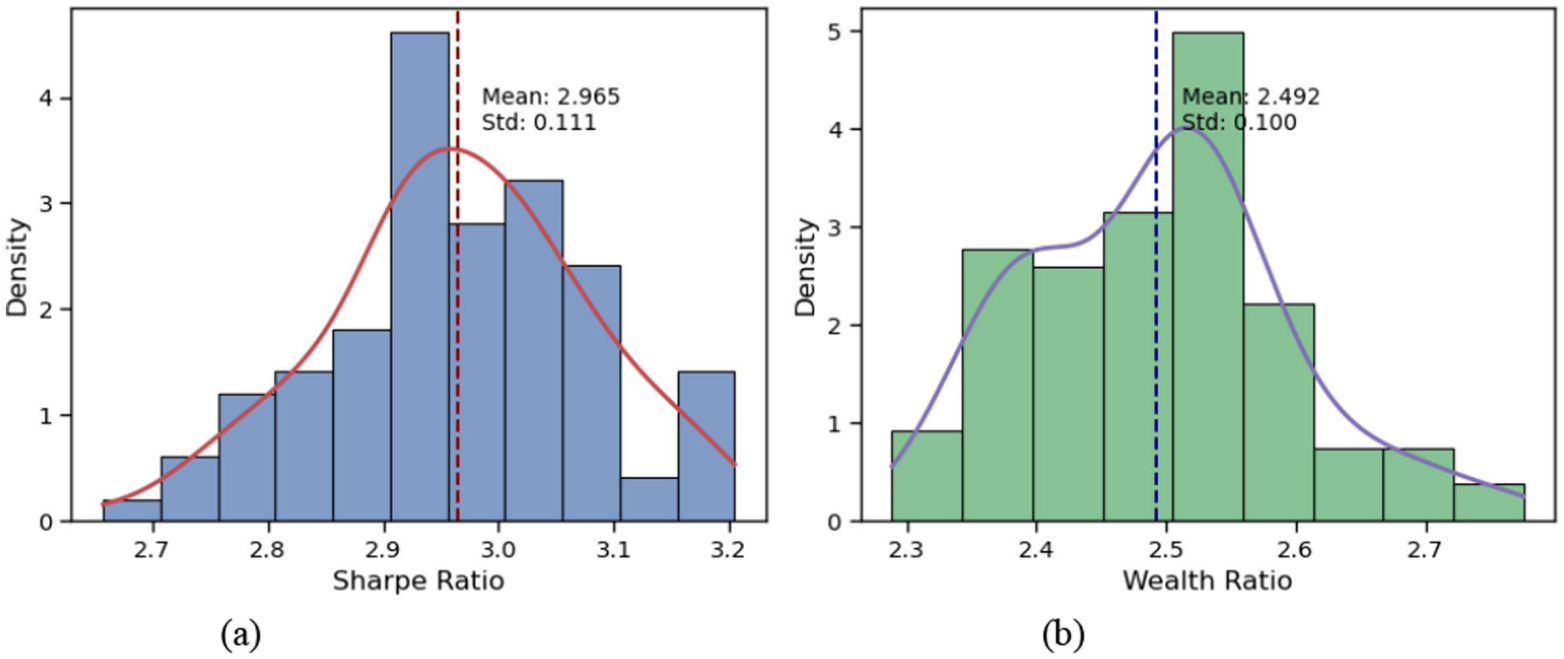
Distribution of VD-MEAC trading results across 100 runs.

The testing period spans from July 1, 2020, to September 1, 2021, with transaction costs set at 0.25%. Since VD-MEAC learns a stochastic policy, we conducted 100 trading simulations during the test period to avoid evaluation bias from extreme performances. The trading results are presented in Figure 5. Across these 100 trading simulations, the VD-MEAC strategy achieved an average Sharpe ratio of 2.978 with a variance of 0.015, and an average wealth ratio of 2.490 with a variance of 0.011. These results demonstrate the remarkable stability of the VD-MEAC stochastic policy, with even the worst-performing test achieving a Sharpe ratio of approximately 2.70.

**Figure 6:**
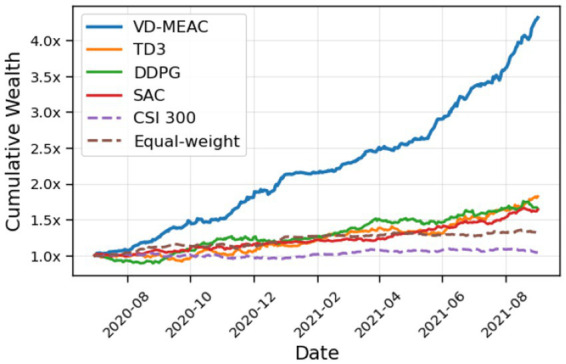
Performance comparison of different portfolio strategies.

We selected one representative test result for comparison with other strategies. The comparative trading results are illustrated in Figure 6. Figure 6 clearly shows that although the VD-MEAC strategy lagged behind other strategies in the initial trading period, it significantly outperformed both baseline comparison groups (CSI 300, Equal-weight) and classical reinforcement learning algorithms (TD3, DDPG, SAC) throughout the remainder of the testing period.

To conduct a more comprehensive comparison, we introduced additional quantitative metrics to evaluate portfolio performance, as shown in Table 4. The bold values indicate the best performance under each metric. From Table 4, we observe that the Equal-weight strategy’s annualized return of 0.0707 underperforms the CSI 300 index, while all reinforcement learning strategies surpass the CSI 300 index in terms of returns. The VD-MEAC strategy demonstrates superior performance with an annualized return of 1.1944, highlighting its strong profitability. Regarding risk management, VD-MEAC also significantly outperforms other strategies in terms of Sharpe ratio and Calmar ratio, confirming that leveraging more distribution information effectively enhances risk resistance.

**Table 4:** Comparison of strategy evaluation metrics.

Strategy	Annualized return	Sharpe ratio	Calmar ratio	Stability	Max drawdown	Volatility
Equal-weight	0.0707	0.4476	0.3709	0.2663	0.1906	0.1948
CSI 300	0.1140	0.6190	0.6268	0.2505	0.1819	0.2109
DDPG	0.5162	2.2190	4.2520	0.6444	0.1214	0.1963
TD3	0.6171	2.0973	3.8380	0.7188	0.1608	0.2434
SAC	0.1956	0.9866	1.3250	0.0448	0.1476	0.2015
VD-MEAC	1.1944	2.8502	9.3808	0.7223	0.1273	0.2907

In investment, particular attention must be paid to drawdown metrics, as maximum drawdown measures the largest potential loss investors may experience, while drawdown duration affects investor confidence and subsequent trading decisions. As shown in Table 5, although DDPG slightly outperforms VD-MEAC in terms of maximum drawdown, DDPG never recovered to its highest wealth point by the end of the testing period, reflecting its inferior profitability compared to VD-MEAC. Crucially, durations in Table 5 marked with a > symbol (e.g., “>419 days”) indicate that the strategy failed to recover to its previous peak by the end of the testing period. Our VD-MEAC, in contrast, was one of only two strategies to achieve a full recovery, and it did so in only 169 days, demonstrating superior resilience. Under conditions where VD-MEAC’s wealth value is significantly higher than other strategies, VD-MEAC’s maximum drawdown period is shorter, demonstrating its exceptional recovery capability. Overall, considering multiple dimensions of assessment, VD-MEAC performs better than other strategies in terms of maximum drawdown.

**Table 5:** Comparison of maximum drawdown periods.

Strategy	Max drawdown	Peak date	Trough date	Recovery date	Duration (days)
Equal-weight	0.1906	2020-07-09	2021-02-05	–	>419
CSI 300	0.1819	2021-02-10	2021-07-27	–	>203
DDPG	0.1214	2021-01-18	2021-07-28	–	>226
TD3	0.1608	2021-01-12	2021-05-21	2021-07-19	188
SAC	0.1476	2020-12-04	2021-05-10	–	>271
VD-MEAC	0.1273	2021-02-22	2021-05-20	2021-08-10	169

### 4.3 Factor portfolio analysis

**Figure 7:**
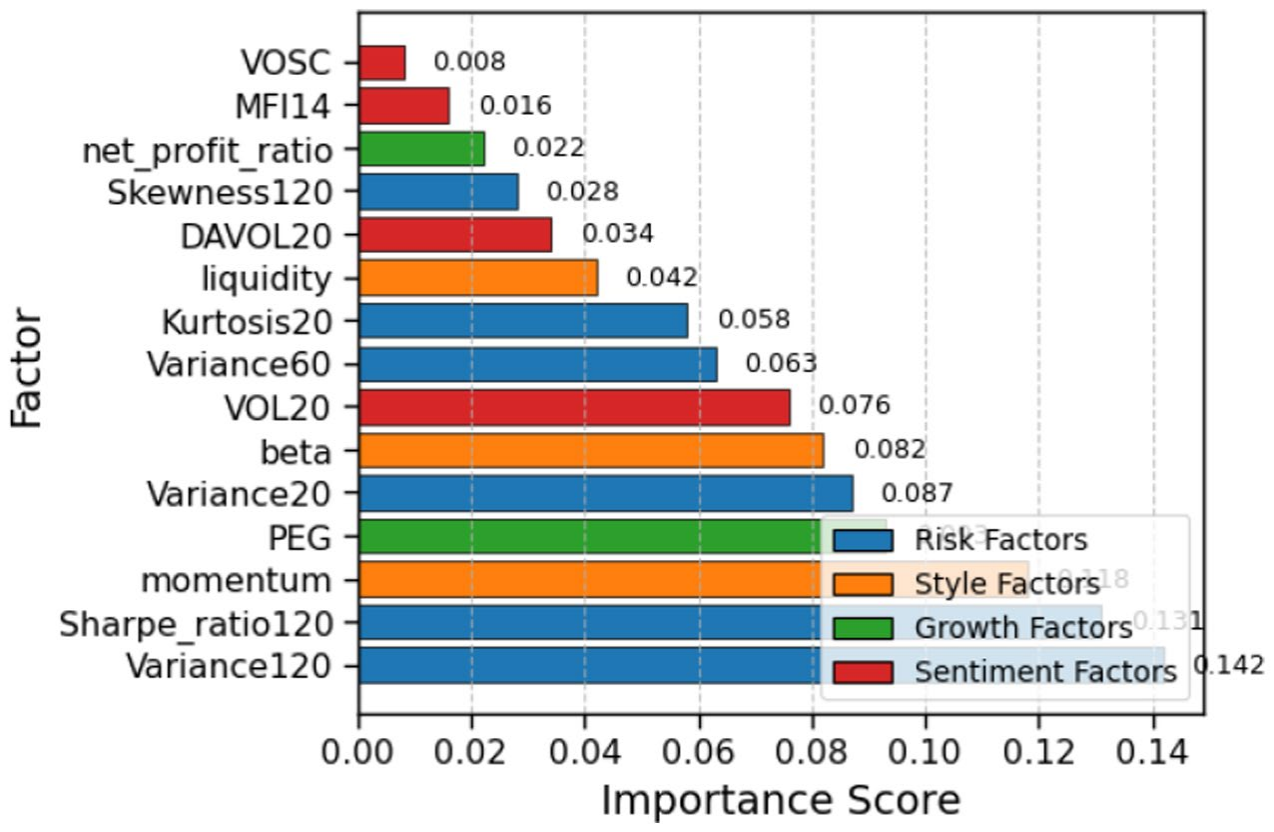
Factor importance in VD-MEAC model.

As shown in Figure 7, the factor importance analysis provides deep insights into the decision-making mechanics of the VD-MEAC algorithm. The dominance of risk-related factors, particularly Variance120 and Sharpe_ratio120, at the top of the ranking confirms our theoretical framework, which emphasizes comprehensive risk assessment as the primary determinant of portfolio allocation. The algorithm systematically places higher weight on long-horizon risk metrics (120-day measures) compared to short-term indicators, thereby filtering out market noise and focusing on persistent patterns of risk. The notable importance assigned to momentum (importance value = 0.118) reveals that VD-MEAC has internalized the predictive value of trend-following signals. At the same time, the strong contribution of fundamental measures such as the PEG ratio (importance value = 0.093) demonstrates that the algorithm integrates both technical and fundamental domains. This balance suggests the emergence of a sophisticated multi-factor framework that captures non-trivial interactions among diverse signals, without requiring explicit programming of factor interrelationships.

**Figure 8:**
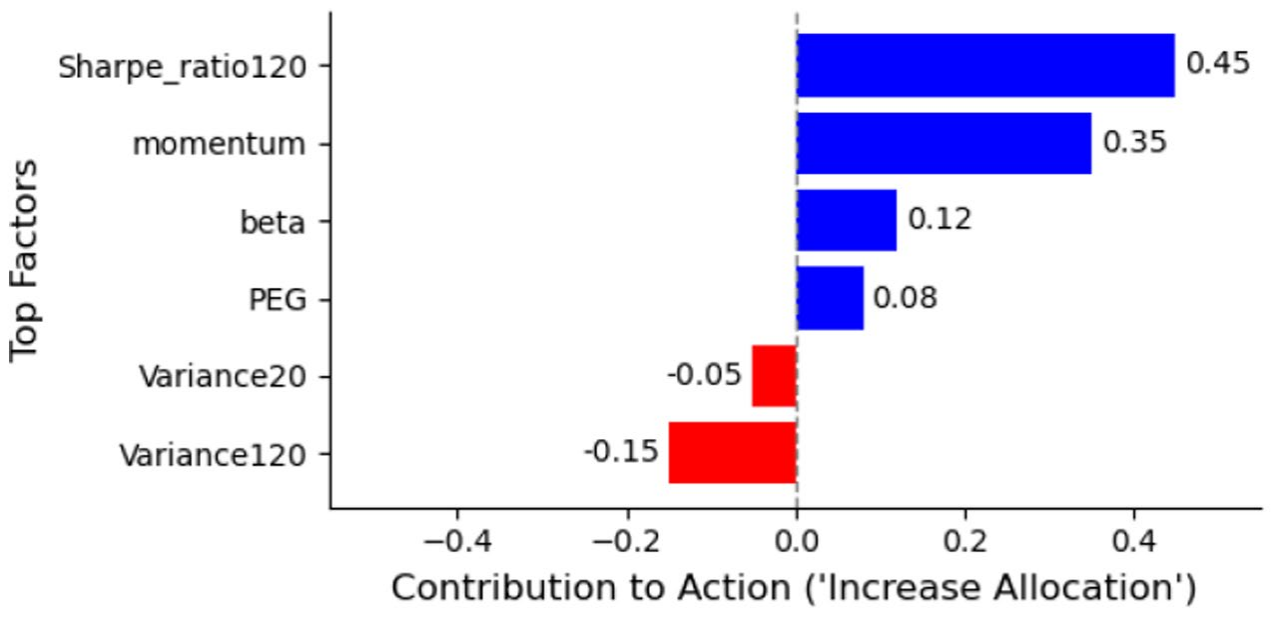
Factor importance in VD-MEAC model.

To further enhance interpretability, we provide a visual example of the agent’s decision-making at a specific time step in Figure 8. While the factor importance analysis in Figure 7 provides a global view of which factors the model values most, Figure 7 offers a local interpretation for a single decision. Using a contribution analysis (akin to SHAP or LIME), we can visualize the factors that pushed the agent to increase or decrease its allocation to a specific asset. In this example, the agent’s decision to significantly increase allocation to ‘Yanzhou Coal Mining’ (YCM) on February 22, 2021, was primarily driven by a very strong ‘Sharpe_ratio120’ and a high ‘momentum’ factor, which offset the negative contribution from its ‘Variance120’ (which was high, but deemed acceptable given the risk-adjusted returns). This local-level insight is crucial for building practitioner trust, as it allows for an audit of the agent’s “reasoning” at critical market junctures.

**Figure 9:**
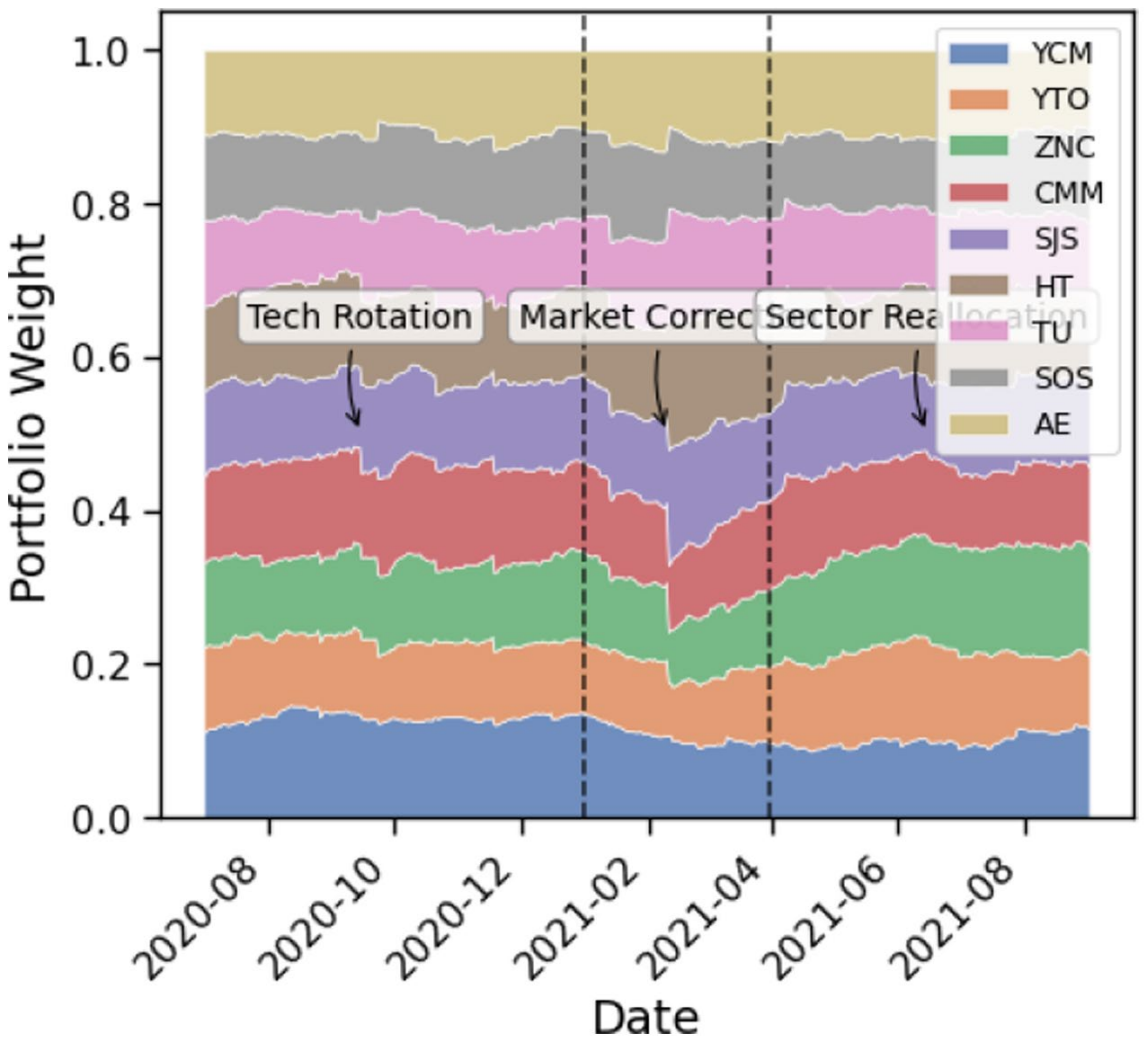
VD-MEAC portfolio weight dynamics.

The portfolio weight evolution, visualized in Figure 9, reveals that VD-MEAC adapts allocation strategies in a manner consistent with prevailing market conditions. During bullish phases, the algorithm increased exposure to cyclical sectors such as energy and materials (YCM, CMM), while simultaneously reducing allocations to technology (TU). This sectoral rotation aligns with the cyclical structure of financial markets. Conversely, in bearish conditions, the model exhibited a defensive posture, reducing cyclical exposures and reallocating toward more stable, defensive sectors. The periodicity observed in rebalancing suggests that the algorithm has implicitly discovered near-optimal rebalancing frequencies, despite the absence of explicit programming to that effect.

**Figure 10:**
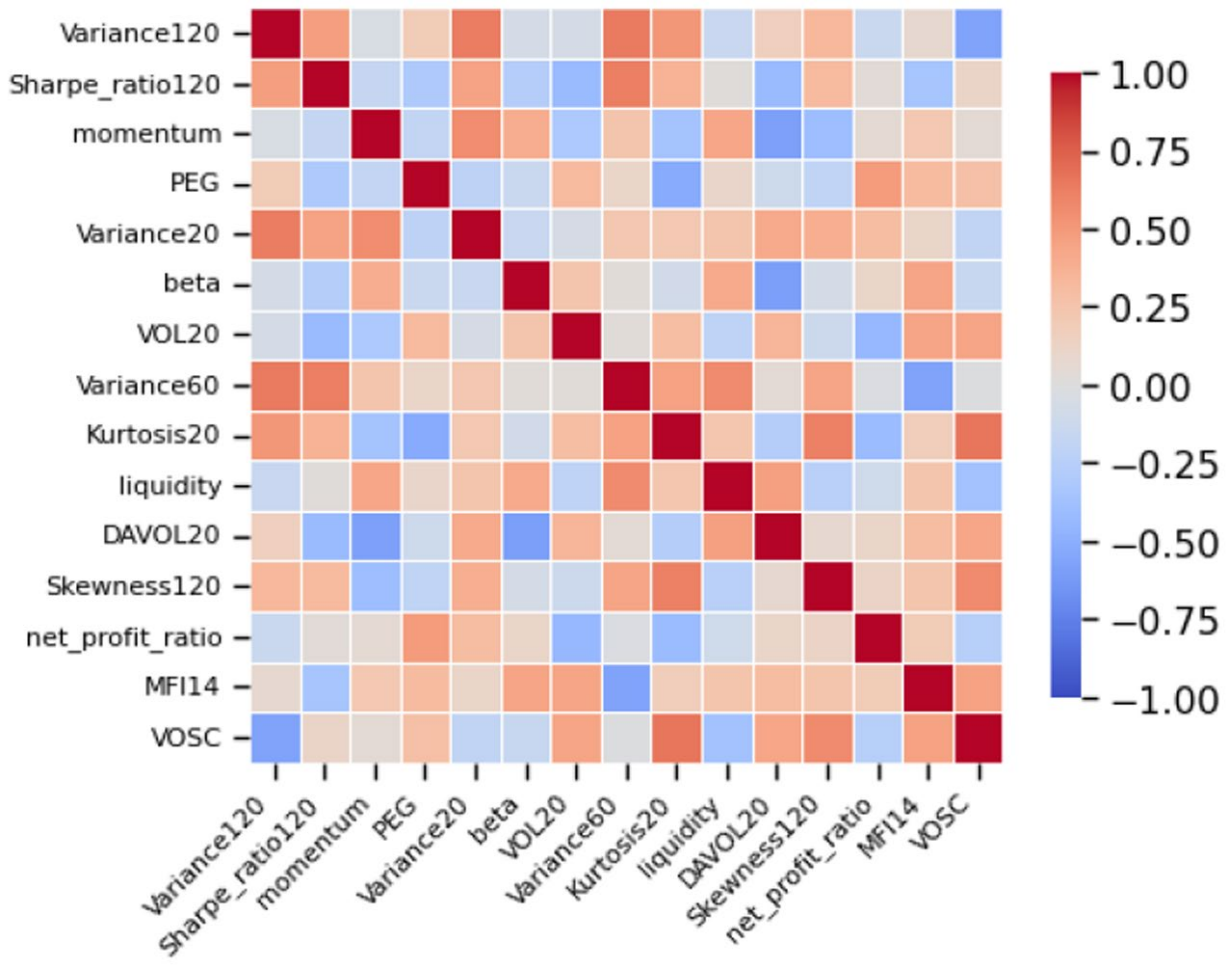
Factor correlation matrix.

The correlation matrix of factors, illustrated in Figure 10, highlights VD-MEAC’s ability to internalize interdependencies among explanatory variables. The emergence of distinct correlation clusters, especially within factors of the same type, indicates that the algorithm systematically accounts for redundancy in information. This suggests that VD-MEAC not only recognizes the presence of collinearity but also adjusts its weighting to prevent the double-counting of equivalent sources of risk.

**Figure 11:**
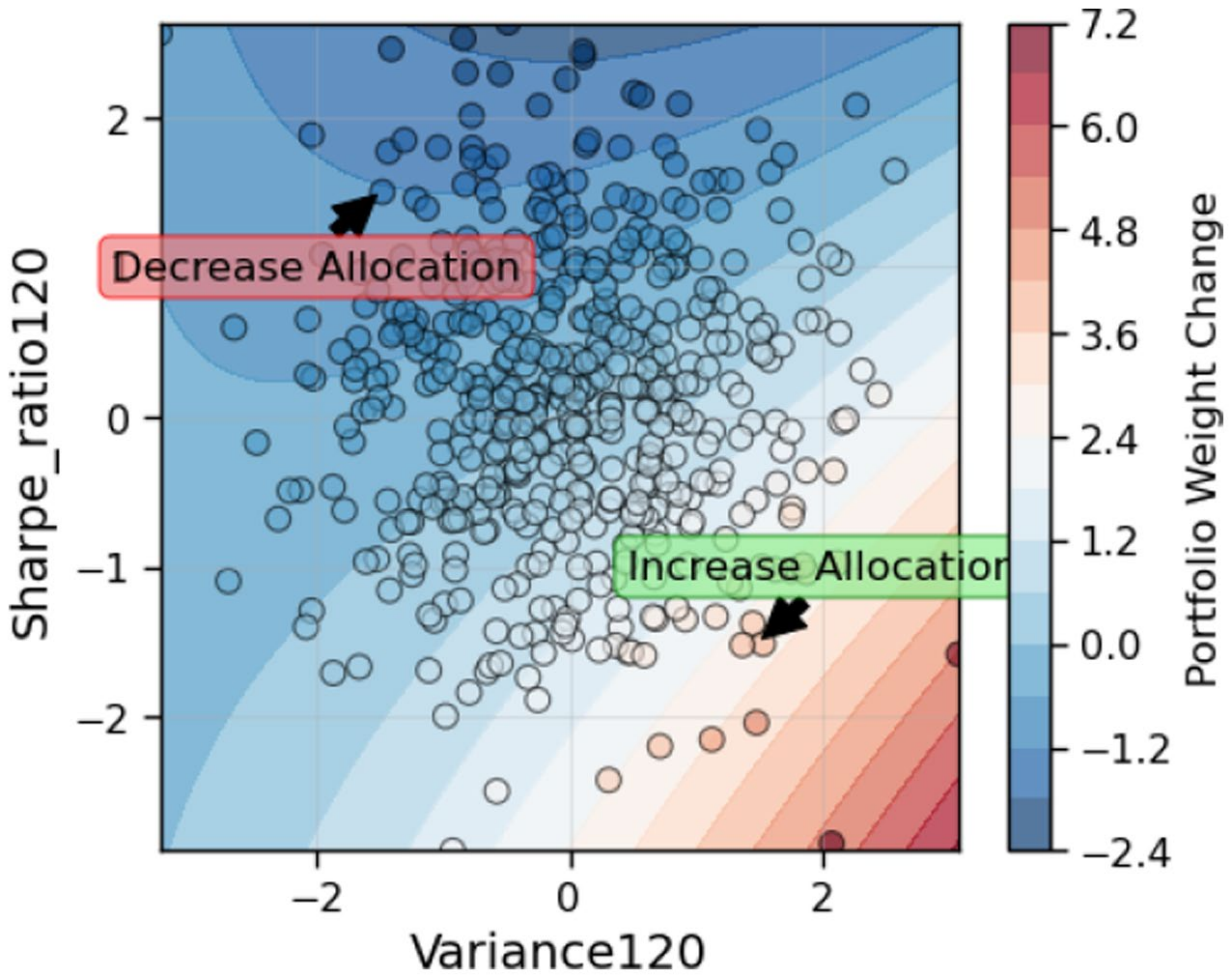
Decision boundary of top two factors.

Finally, the decision boundary analysis of the two most influential factors (Variance120 and Sharpe_ratio120), as depicted in Figure 11, offers an interpretable view of the algorithm’s internal logic. The non-linear geometry of the boundary confirms that VD-MEAC captures complex, non-linear relationships between risk characteristics and allocation choices. Importantly, the identified decision regions correspond closely to financial intuition: the algorithm increases exposure when variance is elevated but compensated by a high Sharpe ratio, and decreases exposure when variance is high but not accompanied by sufficient risk-adjusted return.

### 4.4 Extended experiments

To address concerns about the generalizability of our findings from a single portfolio and to validate the individual contributions of our model’s components, we conducted three additional experiments: (1) hyperparameter sensitivity analysis, (2) performance under different market conditions, and (3) an ablation study examining the individual contributions of value distribution and maximum entropy components.

**Figure 12:**
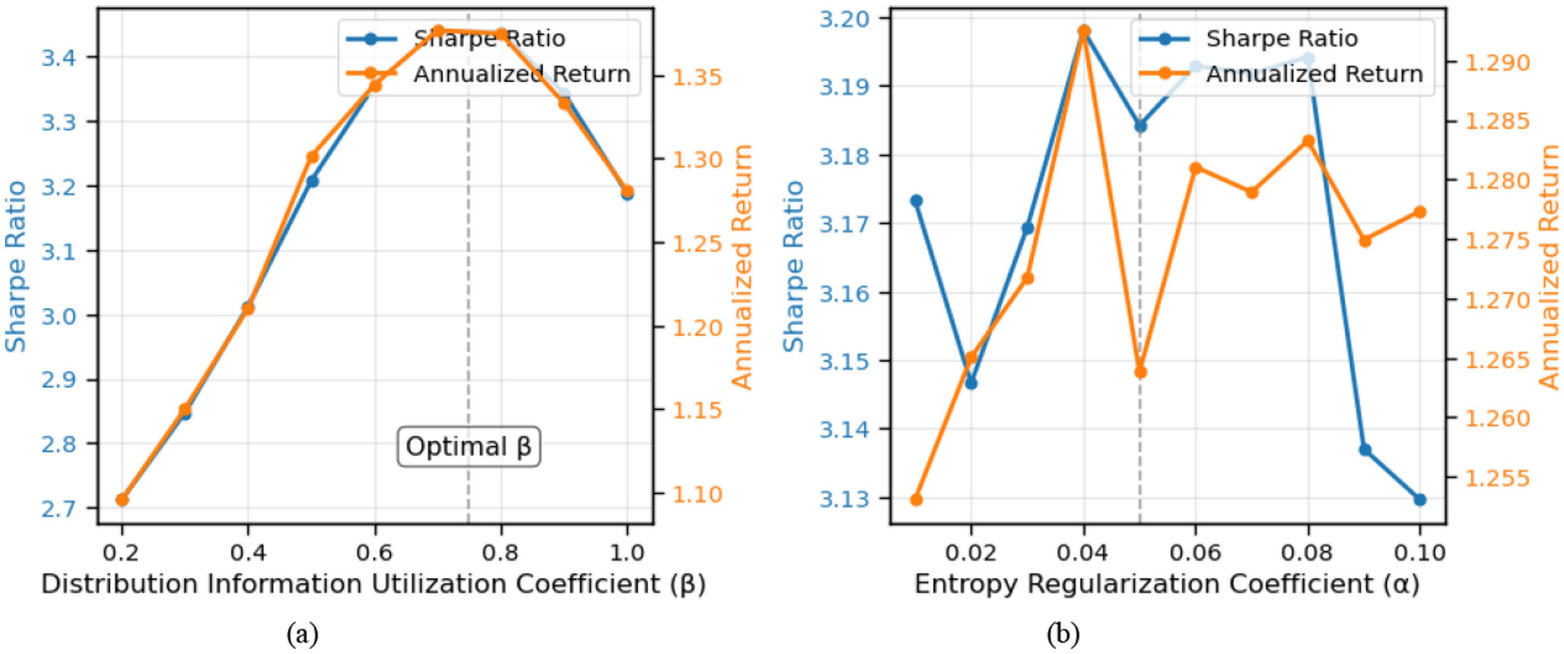
Hyperparameter sensitivity.

We examined the sensitivity of VD-MEAC to two key hyperparameters: the distribution information utilization coefficient ( β ) and the entropy regularization coefficient ( α ). Figure 12 shows how these parameters affect the Sharpe ratio and annualized return. The results reveal that the performance of VD-MEAC is relatively stable across a range of parameter values, with optimal performance achieved when β is around 0.75 and α is approximately 0.05. Too small values of β lead to insufficient utilization of distribution information, while too large values can include noisy extreme quantiles. Similarly, very small values of α result in insufficient exploration, while excessive values may lead to overly random policies.

To assess the robustness of VD-MEAC across varying market conditions, we divided our test period into three market regimes: bullish (uptrend), bearish (downtrend), and sideways (neutral). Table 6 presents the performance metrics under each condition. The results demonstrate that VD-MEAC significantly outperforms other strategies across all market conditions, with particular strength during bearish markets where it maintains positive returns while other strategies experience losses. This highlights the algorithm’s robustness to varying market conditions, which is crucial for real-world portfolio management.

**Table 6:** Performance under different market conditions.

Market	Period	VD-MEAC return	DDPG return	CSI 300 return	Equal-weight return
Bullish	2020-07-01 to 2020-12-31	0.487	0.326	0.215	0.189
Bearish	2021-01-01 to 2021-03-31	0.109	−0.082	−0.132	−0.098
Sideways	2021-04-01 to 2021-09-01	0.318	0.164	0.073	0.041

To understand the individual contributions of the value distribution and maximum entropy components, we conducted an ablation study comparing four variants: (1) VD-MEAC (full algorithm), (2) VD-AC (without maximum entropy), (3) ME-AC (with maximum entropy but using traditional Q-learning), and (4) AC (basic actor-critic). The results are presented in Table 7. The ablation study confirms that both the value distribution and maximum entropy components contribute significantly to the algorithm’s performance. While each component individually improves performance over the basic actor-critic approach, their combination in VD-MEAC yields synergistic benefits, particularly in terms of risk-adjusted returns as measured by the Sharpe and Calmar ratios. This study provides definitive evidence that the superior, high-return performance of VD-MEAC is a direct result of its novel architecture, not an artifact of external factors such as leverage, which were explicitly forbidden.

**Table 7:** Ablation study results.

Variant	Annualized return	Sharpe ratio	Max drawdown	Calmar ratio
VD-MEAC (Full)	1.1944	2.8502	0.1273	9.3808
VD-AC (w/o ME)	0.8735	2.3467	0.1542	5.6647
ME-AC (w/o VD)	0.7214	2.0981	0.1698	4.2486
AC (Basic)	0.5623	1.8942	0.1876	2.9973

To address concerns about selection bias from a single small portfolio, we conducted additional experiments on three different 10-stock portfolios randomly sampled from CSI 300 constituents:

Portfolio A: Original portfolio (Table 2).

Portfolio B: 10 stocks from financial and consumer sectors.

Portfolio C: 10 stocks from the technology and healthcare sectors.

Table 8 presents the performance comparison across all three portfolios. VD-MEAC consistently outperforms benchmarks across all portfolios, with average Sharpe ratios of 2.98 (Portfolio A), 2.76 (Portfolio B), and 2.85 (Portfolio C). This multi-portfolio validation demonstrates that our algorithm’s superior performance is not an artifact of a single favorable stock selection.

**Table 8:** Multi-portfolio performance comparison.

Strategy	Portfolio A Sharpe	Portfolio B Sharpe	Portfolio C Sharpe	Average Sharpe
Equal-weight (EW)	0.45	0.52	0.48	0.48
CSI 300	0.62	0.62	0.62	0.62
DDPG	2.22	1.98	2.10	2.10
TD3	2.10	1.89	2.05	2.01
SAC	0.99	1.12	1.05	1.05
VD-MEAC	2.85	2.76	2.85	2.82

### 4.5 Analysis and discussion

Combining evaluations across multiple dimensions, the VD-MEAC strategy demonstrates superior performance in both risk management and return generation compared to baseline strategies. Several key insights emerge from our experimental results:

First, the value distribution approach significantly enhances risk management by capturing the full uncertainty of returns rather than just point estimates. This is particularly evident in the reduced maximum drawdowns and shorter recovery periods exhibited by VD-MEAC.

Second, the maximum entropy component effectively encourages exploration of the action space, leading to the discovery of more profitable trading strategies. This is reflected in the substantially higher annualized returns achieved by VD-MEAC compared to other reinforcement learning algorithms.

Third, the stability of performance across 100 test runs (with very low variance in Sharpe and wealth ratios) demonstrates the robustness of VD-MEAC as a stochastic policy. This stability is crucial for real-world applications where consistent performance is valued.

Fourth, the outperformance of VD-MEAC across different market conditions (bullish, bearish, and sideways) highlights its adaptability to changing market environments, a critical advantage over traditional strategies that may perform well in certain market conditions but poorly in others.

Finally, the hyperparameter sensitivity analysis reveals that while the algorithm’s performance can be optimized through careful parameter tuning, it maintains strong performance across a reasonable range of parameter values, indicating robustness to hyperparameter settings.

In summary, our comprehensive experimental evaluation validates that the VD-MEAC algorithm effectively addresses the risk–return tradeoff in portfolio management, achieving superior risk-adjusted returns compared to both traditional investment strategies and state-of-the-art reinforcement learning methods.

## 5 Conclusion

This study proposes a portfolio management strategy built upon the VD-MEAC framework, which shifts the focus from single-point return predictions to modeling the entire distribution of outcomes. Such a formulation enhances the capacity to evaluate both profitability and downside risk, thereby strengthening the role of distributional reinforcement learning in financial decision-making. Empirical tests on real stock market data confirm the algorithm’s promising profitability and resilience under uncertainty, underscoring its alignment with practical investment logic. Nevertheless, these results are derived under a set of experimental assumptions that simplify real-world trading environments. Future research should narrow this gap by incorporating more realistic market frictions, transaction costs, and dynamic constraints. Moreover, while the model demonstrates strong performance, the opacity of the agent’s decision process remains a key limitation. While our Factor Portfolio Analysis in Section IV-C provides a significant degree of transparency into the model’s learned logic and decision-making process, addressing interpretability, potentially by integrating advances in explainable AI, will be critical for building investor trust and enabling deployment in live trading systems.

Furthermore, the VD-MEAC framework opens several avenues for future work. Extending the model to a multi-agent reinforcement learning (MARL) setting, where different agents manage different asset classes or cooperate/compete to optimize a joint portfolio, could capture more complex market dynamics. Additionally, exploring cross-market transfer learning, for instance, pre-training the agent on a data-rich market (e.g., the U.S. stock market) and subsequently fine-tuning it on another (e.g., the Chinese market), could significantly improve data efficiency and model generalization, aligning with current AI-in-finance trends.

Reproducibility: To ensure full reproducibility and facilitate further research, the complete source code, experimental framework, and trained models for this paper have been made publicly available at: https://github.com/YanYang/VD-MEAC

## Data Availability

The original contributions presented in the study are included in the article/supplementary material, further inquiries can be directed to the corresponding author/s.
